# Large-Scale Analysis of the *Mycoplasma bovis* Genome Identified Non-essential, Adhesion- and Virulence-Related Genes

**DOI:** 10.3389/fmicb.2019.02085

**Published:** 2019-09-13

**Authors:** Christoph Josi, Sibylle Bürki, Sara Vidal, Emilie Dordet-Frisoni, Christine Citti, Laurent Falquet, Paola Pilo

**Affiliations:** ^1^Department of Infectious Diseases and Pathobiology, Vetsuisse Faculty, Institute of Veterinary Bacteriology, University of Bern, Bern, Switzerland; ^2^Graduate School for Cellular and Biomedical Sciences, University of Bern, Bern, Switzerland; ^3^UMR 1225, IHAP, Université de Toulouse, INRA, ENVT, Toulouse, France; ^4^Department of Biology, Faculty of Science and Medicine, Swiss Institute of Bioinformatics, University of Fribourg, Fribourg, Switzerland

**Keywords:** *Mycoplasma bovis*, random transposon mutagenesis, non-essential genes, adhesion, virulence

## Abstract

*Mycoplasma bovis* is an important pathogen of cattle causing bovine mycoplasmosis. Clinical manifestations are numerous, but pneumonia, mastitis, and arthritis cases are mainly reported. Currently, no efficient vaccine is available and antibiotic treatments are not always satisfactory. The design of new, efficient prophylactic and therapeutic approaches requires a better understanding of the molecular mechanisms responsible for *M. bovis* pathogenicity. Random transposon mutagenesis has been widely used in *Mycoplasma* species to identify potential gene functions. Such an approach can also be used to screen genomes and search for essential and non-essential genes for growth. Here, we generated a random transposon mutant library of *M. bovis* strain JF4278 containing approximately 4000 independent insertion sites. We then coupled high-throughput screening of this mutant library to transposon sequencing and bioinformatic analysis to identify *M. bovis* non-essential, adhesion- and virulence-related genes. Three hundred and fifty-two genes of *M. bovis* were assigned as essential for growth in rich medium. Among the remaining non-essential genes, putative virulence-related factors were subsequently identified. The complete mutant library was screened for adhesion using primary bovine mammary gland epithelial cells. Data from this assay resulted in a list of conditional-essential genes with putative adhesion-related functions by identifying non-essential genes for growth that are essential for host cell-adhesion. By individually assessing the adhesion capacity of six selected mutants, two previously unknown factors and the adhesin TrmFO were associated with a reduced adhesion phenotype. Overall, our study (i) uncovers new, putative virulence-related genes; (ii) offers a list of putative adhesion-related factors; and (iii) provides valuable information for vaccine design and for exploring *M. bovis* biology, pathogenesis, and host-interaction.

## Introduction

*Mycoplasma bovis* is one of the major causative agents of bovine mycoplasmosis. This bacterium is emerging in industrialized countries, where it leads to high economic losses in the dairy and beef industries (Nicholas, 2011; Bürki et al., 2015). Currently, no effective vaccine is available and treatment with antibiotics is hardly efficient (Nicholas, 2011; Gautier-Bouchardon et al., 2014; Perez-Casal et al., 2017). Thus, the development of therapeutic and prophylactic measures to combat bovine mycoplasmosis is an ongoing challenge and requires in-depth understanding of the molecular mechanisms of pathogenicity of this bacterium (Calcutt et al., 2018).

Over the past years, some virulence factors of *M. bovis* have been described (Bürki et al., 2015). The most investigated ones are the variable surface lipoproteins (Vsps) that were shown to be involved in adhesion, antigenic variation, and immune evasion (Sachse et al., 1993, 1996, 2000; Rosengarten et al., 1994; Thomas et al., 2003; Buchenau et al., 2010). Four additional adhesion factors were characterized: the α-enolase (Song et al., 2012), the VpmaX protein (Zou et al., 2013), a NADH oxidase (NOX), which is also involved in H_2_O_2_ production (Zhao et al., 2017) and the TrmFO protein (Guo et al., 2017). Furthermore, two nucleases, the homologs MBOVPG45_0215 and MnuA, and the secretory nuclease MBOV_RS02825, were shown to be associated with cytotoxicity or with the degradation of neutrophil extracellular traps (Sharma et al., 2015; Zhang et al., 2016; Mitiku et al., 2018).

For a long time, the functional characterization of genes in mycoplasmas was hampered by the lack of genetic tools. Since mycoplasmas lack substantial parts of DNA recombination and repair mechanisms (Rocha et al., 2005), the frequency of successful targeted mutagenesis through homologous recombination is very low (Renaudin et al., 2014). For this reason, the fast generation of mutants by the use of transposons is widely used in the field of mycoplasmas (Hedreyda et al., 1993; Hutchison et al., 1999; Dybvig et al., 2000). Random transposon mutagenesis is now available in *M. bovis* (Chopra-Dewasthaly et al., 2005; Sharma et al., 2014). This approach has already led to the identification of the function of some genes in *M. bovis* (Sharma et al., 2015; Rasheed et al., 2017; Zhao et al., 2017). In the closely related species *M. agalactiae*, this technique was used to investigate growth phenotypes of individual or pools of mutants (Baranowski et al., 2010; Hegde et al., 2016).

Transposon mutagenesis has also been useful in identifying a number of mycoplasma genes essential for growth in culture media. In previous studies using other *Mycoplasma* spp. transposon mutant libraries with ≥1000 insertions/genome were built to identify essential genes for growth in rich medium (Glass et al., 2006; French et al., 2008; Dybvig et al., 2010; Lluch-Senar et al., 2015). Moreover, dispensable genes of *M. bovis* were previously identified by direct sequencing of several mutants (Sharma et al., 2014). Yet, a universal and consensual definition of essentiality versus non-essentiality is lacking (Hutchison et al., 2016). Approaches using random transposon mutagenesis take into account individual genes (Hutchison et al., 2016). Non-essentiality and essentiality assignment of genes with transposon mutagenesis is based on the observation of whether an individual gene tolerates disruptions by a transposon (Glass et al., 2006; French et al., 2008; Dybvig et al., 2010; Sharma et al., 2014; Lluch-Senar et al., 2015). Thus, mutagenesis of single genes does not allow us to predict genes that could be simultaneously dispensable or mutants that are mutually exclusive (Hutchison et al., 2016). Moreover, the definition of essentiality versus non-essentiality is dependent on several factors including the selective environment, bacterial growth conditions, and makes use of an artificial environment potentially neglecting differences present in *in vivo* situations (Acevedo-Rocha et al., 2013; Tseng et al., 2013; Hegde et al., 2016; Hutchison et al., 2016).

In this study, we performed a large-scale analysis of the genome of *M. bovis* strain JF4278 to identify non-essential, adhesion- and virulence-related genes in rich culture medium and cell culture conditions. To achieve this aim, we coupled a high-throughput adhesion screening of a random transposon mutant library of *M. bovis* to bioinformatic analyses. Selected individual mutants were then tested for adhesion to confirm previous screening results.

## Materials and Methods

### Bacterial Strain and Growth Conditions

*Mycoplasma bovis* strain JF4278 was isolated from the milk of a cow with pneumonia and mastitis in Switzerland in 2008 (Aebi et al., 2012). JF4278 was grown at 37°C in SP4 medium (Freundt, 1983) supplemented with 50 μg/mL cefoxitin sodium salt (Sigma–Aldrich, Buchs, Switzerland) for 24 h in broth medium or for 3–7 days on agar plates. For the growth of the transposon mutants, 15 μg/mL tetracycline hydrochloride (Sigma–Aldrich, Buchs, Switzerland) was added to the SP4 medium supplemented with cefoxitin. Mycoplasma cell concentrations were defined by CFU counting on agar plates using a stereomicroscope.

### Generation of a Random Transposon Mutant Library

A mutant library of JF4278 was generated by random transposon mutagenesis using a modified version of transposon Tn*4001* (mini-Tn) inserted in the plasmid pMT85-Tet (Chopra-Dewasthaly et al., 2005; Zimmerman and Herrmann, 2005; Dordet Frisoni et al., 2013). *M. bovis* strain JF4278 was transformed using high concentrations of polyethylene glycol 8000 (PEG 8000; Sigma–Aldrich, Buchs, Switzerland) based on a protocol published for the transformation of *Ureaplasma parvum* (Aboklaish et al., 2014). Transformed *M. bovis* were plated on SP4 agar plates supplemented with cefoxitin and tetracycline. After 5–6 days single colonies were picked and suspended in 150 μL SP4 broth with cefoxitin and tetracycline in 96-well plates (TPP^®^, Trasadingen, Switzerland). The cultures were incubated for 24–48 h at 37°C. A total of approximately 3500–4000 individual mutants were collected and individually stored in 96-well plates. Pools of mutants were made from each plate by mixing 10 μL of individually grown mutants. These pools were combined in a single tube to collect all mutants, which were subsequently split into 1 mL aliquots and stored at −80°C.

### Primary Bovine Mammary Gland Epithelial Cells

Primary bovine mammary gland epithelial cells (bMec) were isolated from mammary gland tissues of cows directly after slaughter and cultured as previously described (Wellnitz and Kerr, 2004; Zbinden et al., 2015; Josi et al., 2018). No ethics approval was needed because primary cells were collected from organs of bovine carcasses at the slaughterhouse in accordance with the Swiss Federal Animal Protection Law, RS455. For experiments, cells were routinely seeded in T75 cell culture flasks or 24-well plates (TPP^®^, Trasadingen, Switzerland) and grown until confluency. Twenty hours before infection with *M. bovis*, the medium was changed to minimal essential medium (MEM)-Earle medium without addition of antibiotics but supplemented with 2.2 g/L NaHCO_3_ (Biochrom, Berlin, Germany), 7% fetal bovine serum, and 1% L-glutamine (Biochrom, Berlin, Germany). At the time of infection, cell density reached approximately 1 ^∗^ 10^7^ and 2.25 ^∗^ 10^5^ cells per T75 flask or 24-well, respectively. For infection experiments, cell passages 3–5 were used.

### Screening of the Mutant Library Using an Adhesion Assay and DNA Extraction

**FIGURE 1:**
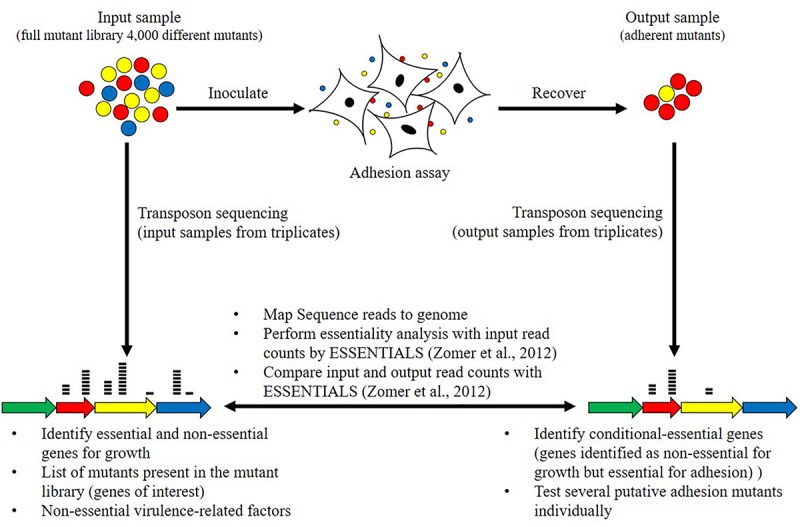
Schematic representation of the adhesion screening and bioinformatics analyses. The adhesion screening was performed with the full mutant library containing approximately 4000 mutants (input sample). After adhesion, adherent mutants (output sample) were recovered. The screening of the same full random transposon mutant library was performed in three replicates, leading to the collection of three input samples and three output samples. Transposon sequencing was carried out by parallel sequencing of the regions flanking each transposon to identify disrupted genes. Transposons sequencing read data from input samples from triplicates were used to categorize genes as essential and non-essential for growth on rich medium using the ESSENTIALS software (Zomer et al., 2012). Transposons sequencing read data of input and output samples from triplicates were then compared to identify conditional-essential genes (essential for adhesion) using the ESSENTIALS software (Zomer et al., 2012).

The screening methodology of this study is summarized in Figure 1. A pool of the mutants, containing all collected mutants, was either directly lysed (input sample) or used for an adhesion assay (Figure 1). The full mutant library is referred as the input sample and the mutants recovered after the adhesion experiment are referred as the output sample (Figure 1). The input sample and output sample from one experiment were collected in parallel. The screening of the full random transposon mutant library was performed in three replicates leading to the collection of three input samples and three output samples. The DNA of input samples and output samples was sequenced by transposon sequencing (Figure 1). The sequencing data were processed to identify non-essential, adhesion- and virulence-related genes (Figure 1).

The adhesion assay was adapted in our laboratory from a previous protocol (Sachse et al., 1996; Sachse, 1998; Josi et al., 2018). For one replicate, one aliquot of the pooled complete mutant library was thawed, supplemented with 1 mL fresh SP4 medium, and incubated for 2 h at 37°C. Mycoplasma cultures were washed once and suspended in buffer A (0.05 M Tris–HCl, pH 7.2, 0.1 M NaCl, and 1 mM CaCl_2_). Before infection, bMec were washed with buffer A and 6 mL buffer A was added in the T75 flask. Approximately 5 ^∗^ 10^7^ mycoplasmas were added to infect bMec. *M. bovis* was allowed to adhere to bovine cells for 30 min on a rocker with 22 strokes/min and an amplitude of 3.5 cm at 37°C. bMec were washed three times with 15 mL PBS (Thermo Fisher Scientific) and manually rocked 30 times to remove unattached mycoplasmas. bMec with attached mycoplasmas were trypsinized and collected in 10 mL MEM-Earle medium. Twenty microliters of the output sample were used to determine the CFU. The remaining sample was centrifuged at 10,000 × *g*. Total DNA of the output sample was isolated using the DNeasy^®^ Blood & Tissue Kit (QIAGEN GmbH, Hilden, Germany) for cultured cells according to the manufacturer’s protocol. To prevent saturation, three columns were used to extract DNA from each experiment. An initial RNase A treatment step was included and DNA from each column was eluted in 150 μL H_2_O and pooled. In parallel, mycoplasmal concentration of the input sample was also determined by CFU counting. The input sample was then centrifuged and lysed with lysis solution [buffer A supplemented with 1% Tween^®^ 20 (Sigma–Aldrich, Buchs, Switzerland) and 0.24 mg/mL Proteinase K (Roche Diagnostics, Rotkreuz, Switzerland)]. The input sample was then heated for 1 h at 65°C and 15 min at 96°C. The percentage of *M. bovis* adherent to bMec was calculated by dividing the CFU calculated in the input sample by the CFU counted in the output sample. The assay was performed in triplicates leading to the collection of three input DNA samples and three output DNA samples. The concentration and purity of the three DNA samples from inputs and outputs were checked with a NanoDrop 1000 Spectrophotometer (Thermo Fisher Scientific) and stored at −20°C for further use.

### DNA Library Preparation for Transposon Sequencing on the Illumina NextSeq Platform

Input and output DNA samples were prepared for transposon sequencing on the Illumina NextSeq Platform. Two major steps were performed: (1) Enrichment of amplicons from the transposon-junctions and depletion of eukaryotic DNA and (2) Amplification of the library to add Illumina adapters. The method is briefly described here but the full and detailed protocol is in Supplementary Table 1. A method for amplicon library preparation was set up involving biotinylated primers specific for the inserted transposon present in *M. bovis* mutants. Purifications were done using streptavidin-coated magnetic beads to limit contamination with eukaryotic DNA in output samples. Amplicons of the transposon-junction sequences were prepared using a non-restrictive linear amplification-mediated PCR (nrLAM-PCR) approach (Gabriel et al., 2009). A previously published protocol was followed (Paruzynski et al., 2010) with the following modifications. Instead of a linker-ligation as described in the study of Paruzynski et al. (2010), a homopolymer-C tail of controlled length was added to linear PCR products needed for subsequent exponential PCRs (Lazinski and Camilli, 2013). The combination of nrLAM-PCR with homopolymer tail-mediated ligation PCR (HTML-PCR) was previously published (Dawoud et al., 2014). Amplicon libraries were sent to Microsynth (Microsynth AG, Balgach, Switzerland) for barcoding and adding Illumina adapters. The six libraries were gel purified, quantified, pooled, and sequenced in the same run with Illumina NextSeq 1 × 75 bp high-output.

### Bioinformatics Analysis

#### Illumina Sequence Data Analysis

Transposon sequencing reads of input and output samples were obtained in FASTQ format. Adapter, primer, and transposon sequences were removed and reads were trimmed to 25 bp using Trimmomatic (Bolger et al., 2014). Raw reads and trimmed reads from input and output samples were deposited in the European Nucleotide Archive (ENA) (Bioproject: PRJEB31021). Quality control of trimmed reads was performed with FastQC and reads were mapped against the *M. bovis* strain JF4278 reference genome (GenBank accession no. NZ_LT578453.1) with Bowtie 2 (Langmead and Salzberg, 2012). Alignment parameters are shown in Supplementary Table 1. BAM files were generated after mapping and filtered for mapping quality ≥12 using BamTools (Barnett et al., 2011). These bioinformatics procedures were performed using the web-based scientific analysis platform Galaxy^1^ (Afgan et al., 2018). Filtered BAM files were used for visualization of the alignments in Geneious version R11.1.2 (Kearse et al., 2012).

#### Essentiality Analysis

Essentiality analysis was performed with ESSENTIALS^2^, which is an open source, web-based software tool for insertion sequencing analysis (Zomer et al., 2012). Trimmed reads from input samples were uploaded as individual control samples from the same library. Reads were aligned directly to the *M. bovis* strain JF4278 reference genome with ESSENTIALS. Count data for read counts per insertion or gene were generated and compared to the expected number of reads per gene calculated by ESSENTIALS. Count data were combined and normalized for differential expression analysis with EdgeR by ESSENTIALS (Robinson et al., 2010; Zomer et al., 2012). Parameters used for the essentiality analysis by ESSENTIALS are shown in Supplementary Table 1.

#### Conditional-Essentiality Analysis

Conditional-essentiality analysis which corresponds to the detection of genes identified as non-essential for growth but essential for adhesion was performed using ESSENTIALS (Zomer et al., 2012). Compared to the essentiality analysis, output samples were included and uploaded as individual target samples from the same library. Input samples were uploaded as individual control samples from the same mutant library. Parameters used for the ESSENTIALS run were kept the same as explained above. In addition to the prediction of adhesion-related factors by ESSENTIALS, detection of putative adhesins with the SPAAN software (Sachdeva et al., 2005) was performed on the predicted *M. bovis* proteome.

#### KEGG Pathway Analysis

Functional KEGG pathway annotation of JF4278-predicted proteins was achieved using the web-based version of KOBAS 3.0 against the annotated genome of *M. bovis* type strain PG45^3^ (GenBank accession no. NC_014760.1) (Wise et al., 2011; Xie et al., 2011). Since KEGG pathway annotation with KOBAS 3.0 was performed for protein sequences only, rRNAs and tRNAs were annotated manually to KEGG pathways via the *M. bovis* PG45 reference genome available on the KEGG database^4^. KEGG pathway enrichment of essential genes compared to the complete gene set of JF4278 was assessed. First, KEGG pathway fold enrichment (fold enrichment of proportion of JF4278 essential genes compared to the proportion of all JF4278 genes belonging to the respective KEGG pathway) was calculated. Secondly, the significance of overrepresentation of the respective KEGG pathway in the essential geneset compared to the complete geneset of JF4278 was calculated using Fisher’s exact test.

#### Comparison Between Essential Genes of JF4278 and Non-essential Genes in *M. bovis* PG45

*Mycoplasma bovis* PG45 non-essential protein-coding genes were blasted in NCBI protein–protein BLAST^5^ to find the respective JF4278 homologs (Sharma et al., 2014). Non-essential genes of PG45 that were found essential in JF4278 were compared to other *Mycoplasma* spp. (French et al., 2008; Dybvig et al., 2010; Lin and Zhang, 2011; Liu et al., 2012). The persistence of these genes in *Mycoplasma* spp. was assessed as described in the study of Liu et al. (2012). Since the latter study did not include *M. bovis* strains *M. agalactiae* strain PG2 (GenBank accession no. NC_009497.1) orthologs to JF4278 were first identified by protein–protein BLAST (Sirand-Pugnet et al., 2007b). Using PG2 orthologs, orthologs to other *Mycoplasma* spp. were then identified based on the study by Liu et al. (2012) and compared to the essential gene set of *M. pulmonis* strain UAB CTIP (French et al., 2008; Dybvig et al., 2010). *M. pulmonis* was selected for comparisons due to its thorough assignment of essential genes and because it belongs to the Hominis group like *M. bovis* (May et al., 2014). Finally, *M. pulmonis* orthologs were analyzed to define if they belong to the putative core set of essential genes in *Mycoplasma* genomes^6^ (Lin and Zhang, 2011).

#### Additional Softwares

To show density distributions of essentiality values obtained with ESSENTIALS, Kernel density plots using an Epanechikov model were generated by a web-based tool^7^. The software TMpred^8^ was used to predict putative transmembrane helices (TM-Hs). Statistical analysis, dot plots, and box plots were made in R version 3.4.3.

### Isolation of Candidate Mutants Involved in Adhesion

Selected mutants identified as conditional-essential in the adhesion assays were recovered from the mutant library by PCR screening using a step-wise approach. PCR primers were designed using the alignments obtained from the transposon sequencing data (Supplementary Table 1). PCRs were performed on the mutant pool of all collected 96-well plates (36 pools), to identify the 96-well plate containing each mutant. Thereafter, PCRs were carried out on pools of each row of the respective plate and of each well within the positive row. The culture obtained from the well assigned to the selected mutant was filtered with a 0.2-μm syringe filter and plated on SP4 agar plates containing tetracycline. A single colony was picked and grown in SP4 liquid medium containing tetracycline. A final PCR and sequencing of the obtained PCR product were performed to confirm the isolation of the selected mutants.

### Adhesion Assay With Selected Mutants

The adhesion assay for the relative quantification of adherent mycoplasmas was adapted from a previous protocol established in our laboratory (Josi et al., 2018). The quantification of adherent *M. bovis* was carried out using real-time qPCR (Rossetti et al., 2010). The adhesion assay was performed for each selected mutant individually as described in the section “Screening of the Mutant Library Using an Adhesion Assay and DNA Extraction” but in a 24-well plate setup. As described previously (Josi et al., 2018), lysates of the *M. bovis*-mutant inoculum and infected cell lysates were collected to quantify the relative amount of adherent mycoplasmas. The percentage of adherent *M. bovis* relative to the amount of *M. bovis* added for the infection was calculated as follows (adapted from Nicholson et al., 2012):

$$\%\mathrm{of}\mathrm{adherent}M.bovis=2^{\left(a-b\right)}*100$$

where *a* = *C*_t_ of mycoplasmas added for infection; and *b* = *C*_t_ of adherent mycoplasmas. The assays were performed in duplicates in four independent experiments. Statistical comparison of each mutant to the wild type (wt) was performed by the two-sample Wilcoxon test.

## Results and Discussion

### Mutant Library Transposon Sequencing Results and Alignment to *M. bovis* Strain JF4278 Reference Genome

The transposon sequencing results from the three replicates of input and output samples are shown in Table 1. Transposon sequencing runs yielded between 39 and 56 million reads for the different input and output samples (Table 1). The transposon sequencing reads were aligned to the *M. bovis* strain JF4278 genome retrieved from NCBI (GenBank accession no. NZ_LT578453.1). The alignment rate was between 43 and 67%, corresponding to between 22 and 28 million aligned reads for the different input and output samples (Table 1). The GC content of unaligned reads was 67.5%. In these reads, the G content increased steadily to nearly 100% toward the end of the read, reflecting sequencing of the added poly-C tail during amplicon library preparation. The remaining specific sequences at the start of unaligned reads were too short to be accurately aligned. On average 26 million transposon sequencing reads per sample could be aligned to the JF4278 reference genome. Therefore, with approximately 4000 independent transposon insertions sites within the JF4278 genome, the average sequencing depth (reads/transposon insertion) of each transposon-junction is approximately 6500 per sample. The size of the *M. bovis* strain JF4278 genome is 1,038,531 bp. With approximately 4000 independent transposon insertions sites within the JF4278 genome, the average density of our mutant library is one insertion event every 260 bp.

**TABLE 1:** Summary of the transposon sequencing results from input and output samples of the adhesion assay.

**Sample name**	**Adhesion assay replicate^1^**	**Total number of transposon sequencing reads (million)**	**Number of reads aligned to JF4278 (million)**	**Alignment rate to JF4278 (%)**
Input 1	1	39	26	67
Output 1	1	46	26	57
Input 2	2	44	28	64
Output 2	2	56	28	50
Input 3	3	47	28	60
Output 3	3	51	22	43
Average	N/A	47	26	55

*^1^Input and output samples from one replicate were collected in parallel.*

**FIGURE 2:**
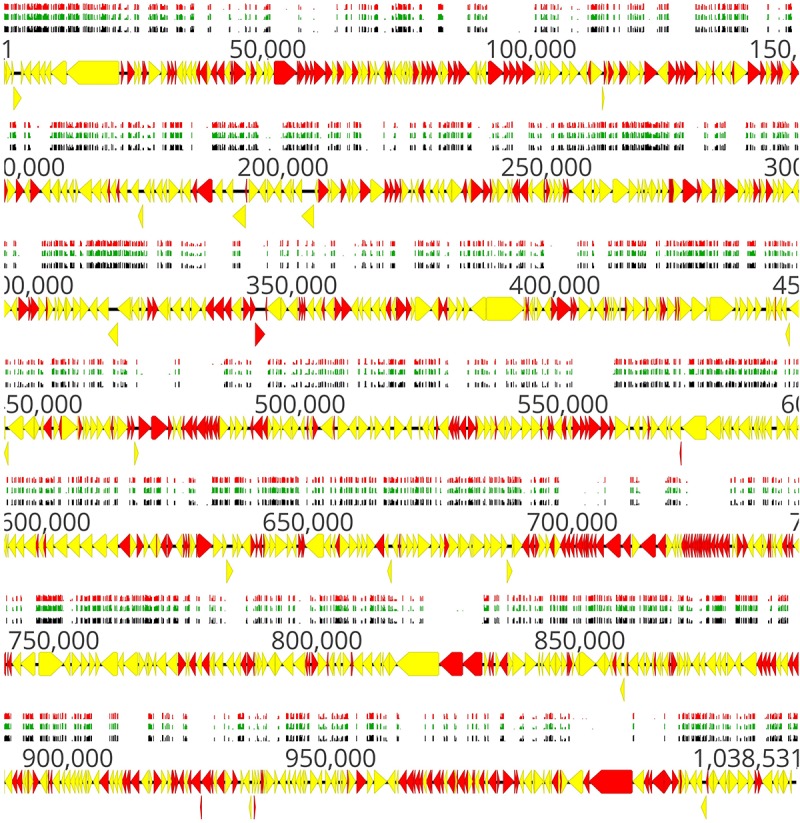
Location of transposon insertion sites in the genome of *M. bovis* strain JF4278. Genes and their orientation are indicated by arrows. Genes found essential with ESSENTIALS are filled in red, while non-essential genes are filled in yellow (Zomer et al., 2012). Mapping of the transposon integrations sites from the transposon sequencing of the full mutant library (input samples) is represented by black, green, and red bars (each corresponding to one replicate experiment). The height of the insertion bars represents the read count per insertion. Approximately 4000 independent transposon insertion sites were detected with an average sequencing depth (reads/transposon insertion) of 6800 per transposon-junction per replicate.

A schematic illustration of the alignment of the transposon sequencing reads from the three replicates of the full mutant library (input samples) is shown in Figure 2. Distribution of transposon insertion sites as well as read counts per transposon insertion (height of bars) among the three replicates of the full mutant library (input samples) is highly similar (Figure 2). The sequencing depth between different transposon insertion sites and the insertion density between different genes varies and this data were used to identify essential and non-essential genes of *M. bovis* strain JF4278 with the software ESSENTIALS (Figure 2). No obvious hot and cold spots for transposon Tn*4001* integration were observed (Figure 2). Compared to integration with Tn*916*, also commonly used in Mollicutes, Tn*4001* is not known to integrate preferably in hot spots in mycoplasmas (Renaudin et al., 2014). However, a preference of secondary insertions of Tn*4001* in regions rich in palindromic and cruciform elements was shown in *M. genitalium* (Glass et al., 2006).

### Essential Genes of *M. bovis* Strain JF4278 Can Be Identified With the Software ESSENTIALS

**FIGURE 3:**
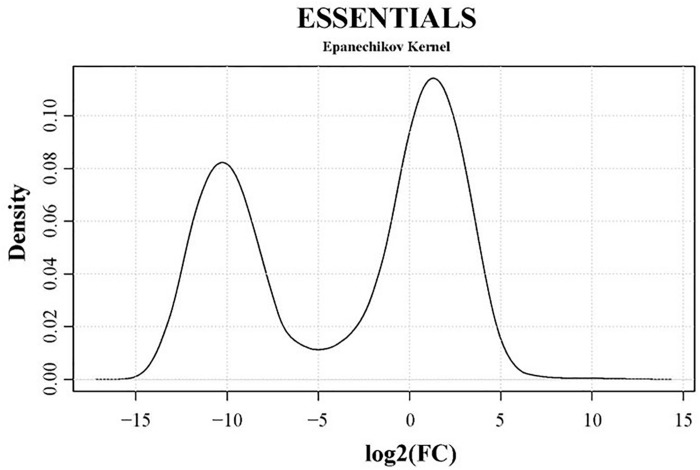
Density plot of essentiality values obtained with the software ESSENTIALS. Read counts per gene of the input samples were compared to the expected number of reads per gene calculated by ESSENTIALS (Zomer et al., 2012). *M. bovis* strain JF4278 genes are assigned a fold change (FC) value of read counts in log2 scale compared to the expected number of reads per gene. Log2(FC) values (essentiality values) for all genes are shown on the *x*-axis of the density plot. Essentiality values show a bimodal distribution. The log2(FC) value of non-essential JF4278 genes is around zero. Genes more likely to be essential have negative log2(FC) values. The cut-off value for essential genes suggested by the software ESSENTIALS for this data set is log2(FC) < –4.64.

Genes essential for growth in SP4 medium were analyzed using the software ESSENTIALS (Zomer et al., 2012). Non-essentiality and essentiality assignment of genes is not based solely on the absence or presence of gene disruption but rather on the amount of disruption tolerated by a gene. Read data from input samples were used for the essentiality analysis. An expected number of reads per gene is automatically calculated by ESSENTIALS. This is based on the size of the used mutant library, the number of insertion sites per gene, and the sequencing depth of the transposon-junctions (Zomer et al., 2012). The obtained read counts per gene of input samples were compared to the expected number of reads per gene calculated by ESSENTIALS (Figure 3). Thereby, genes are assigned a fold change (FC) value of read counts in log2 scale. Log2(FC) values for all JF4278 genes were obtained with ESSENTIALS (Figure 3 and Supplementary Table 2). The log2(FC) values of JF4278 genes show a bimodal distribution in the density plot (Figure 3). The log2(FC) value of non-essential genes is around zero, as the obtained read counts correspond to the expected number of reads per gene (Figure 3). On the other hand, genes with underrepresented read counts [strongly negative log2(FC) values] are more likely to be essential (Figure 3). The log2(FC) value of a gene can be interpreted similarly to a fitness score where significantly underrepresented (adj. *P*-value ≤ 0.05) genes below an automatically calculated log2(FC) cut-off value are considered essential. The log2(FC) cut-off suggested by ESSENTIALS for our dataset was <−4.64 (Figure 3).

According to these criteria [log2(FC) < −4.64 and adj. *P*-value ≤ 0.05], 352 out of 900 genes were found to be essential for growth in SP4 medium (Supplementary Table 2). For 36 out of the remaining 548 non-essential genes, log2(FC) and adjusted *P*-values could not be obtained. These genes mainly encode for proteins with DDE transposase and/or integrase core domain and were present in multiple copies (Supplementary Table 2). Reads from highly repetitive regions or from genes present in multiple copies cannot be assigned to a specific position in the genome and are excluded by the software ESSENTIALS (Zomer et al., 2012). However, if an insertion occurs in a non-repetitive segment of gene containing highly repetitive sequences, this read will be taken into account by the software ESSENTIALS. For this reason, not all DDE transposases are among the 36 genes without log2(FC) and adjusted *P*-values (Supplementary Table 2).

Distribution of transposon insertion sites, as well as the read counts in log10 scale (height of bars) from the three sequencing replicates of the full mutant library (input samples), is in line with the predicted essentiality of genes (Figure 2 and Supplementary Table 2). Genes with no or few transposon insertions and with limited numbers of reads were identified as essential (Figure 2, red arrows), while non-essential genes (Figure 2, yellow arrows) contained more insertions and higher read counts/gene. The high density of essential genes found in the locus between positions nt 679,886 and 729,966 corresponded to genes encoding small ribosomal proteins, ribosomal RNA, and other factors involved in translation (Figure 2 and Supplementary Table 2). Other prominent essential operons contained genes encoding proteins involved in oligopeptide permease (Opp) systems (nt 91,927–100,788) and subunits of the F_1_F_0_ ATPase (nt 484,670–477,651) (Beven et al., 2012).

### Essential Genes Are Involved in Genetic Information Processing and Metabolic Pathways

**FIGURE 4:**
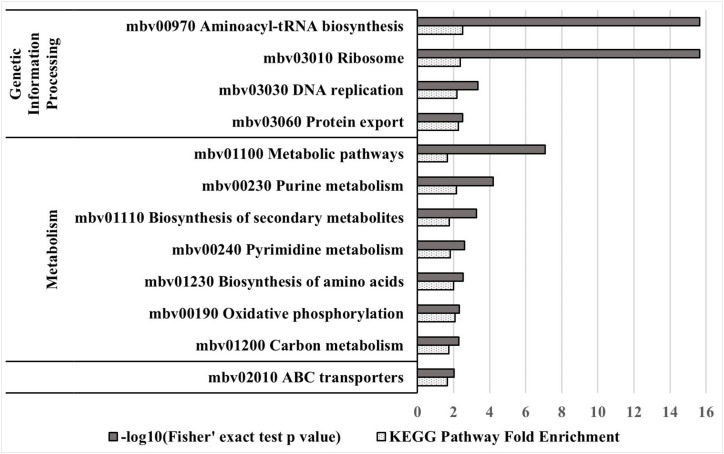
KEGG pathway enrichment analysis of essential genes in strain JF4278. Different KEGG pathways from *M. bovis*, with the corresponding mbov ID number, are displayed. Significant (*P*-value ≤ 0.01) overrepresented KEGG pathways in the essential gene set compared to the complete gene set of strain JF4278 are shown. Gray columns correspond to the *P*-value for significance of enrichment in –log10 transformation. Spotted columns correspond to the fold enrichment of individual KEGG pathways between complete and essential gene set. Significance of enrichment was calculated with the Fisher’s exact test.

Kyoto encyclopedia of genes and genomes (KEGG) pathway enrichment analysis of essential genes of strain JF4278 was performed. Using PG45 as reference genome, 277 genes of strain JF4278 were assigned to one or several of the 52 KEGG pathways (Supplementary Table 3). Two hundred and nine genes out of the 277 were found to be essential with the ESSENTIALS software. First, KEGG pathway fold enrichment (fold enrichment of proportion of JF4278 essential genes compared to the proportion of all JF4278 genes belonging to the respective KEGG pathway) was calculated (Supplementary Table 3). Secondly, the significance of the overrepresentation of the respective KEGG pathway in the set of essential JF4278 genes compared to the complete set of JF4278 genes was calculated using Fisher’s exact test (Supplementary Table 3). Eighteen out of the 52 KEGG pathways were significantly overrepresented (*P*-value ≤ 0.05) in the set of essential genes of strain JF4278 (Supplementary Table 3). KEGG pathways with a highly significant enrichment (*P*-value ≤ 0.01) were examined in more detail (Figure 4). Genetic information processing pathways were among the top hits. Nearly all genes assigned to pathways “Ribosome,” “Aminoacyl-tRNA biosynthesis,” and “DNA replication” were found to be essential in JF4278. Only three genes were assigned to transcription in the pathway “RNA polymerase.” Even though these three genes were found to be essential, no significant enrichment was obtained (Supplementary Table 3). Genes involved in replication and translation belong to the basic cellular machinery and were also found to be essential in many bacterial species (Grazziotin et al., 2015). Furthermore, many genes assigned to metabolic pathways were found to be essential in JF4278 (Figure 2). Among the top hits were genes involved in nucleotide metabolism (purine and pyrimidine metabolism), biosynthesis of secondary metabolites and amino acids. Genes assigned to oxidative phosphorylation encode for subunits of the F_1_F_0_ ATPase found in different *Mycoplasma* spp. However, in *Mycoplasma* spp. the F_1_F_0_ ATPase is involved in the maintenance of the electrochemical gradient by ATP hydrolysis rather than in the generation of ATP (Beven et al., 2012). While carbon metabolism genes were significantly overrepresented in the set of essential JF4278 genes, individual carbohydrate metabolism pathways were not (Figure 4 and Supplementary Table 3). In *Mycoplasma* spp., glycerol derived from host-phospholipids is a major source of carbon and energy (Blötz and Stülke, 2017). In the *M. bovis* strain JF4278, most of the genes assigned to “glycerophospholipid metabolism” were found to be essential (Supplementary Table 3). In *Mycoplasma* spp. many metabolic pathways are incomplete and mycoplasmas strongly depend on host carbon sources and nutrients (Razin et al., 1998; Masukagami et al., 2017). It can be expected that the remaining genes involved in metabolic pathways of mycoplasmas are essential to further metabolize nutrients. In *M. bovis* strain JF4278 the remaining parts of metabolic pathways were indeed found to be essential for growth on rich medium. Furthermore, the high proportion of essential ABC transporters found in the *M. bovis* strain JF4278 genome might reflect the strong dependency of mycoplasmas on nutrients uptake (Figure 4 and Supplementary Table 3).

### Nineteen Non-essential Genes of PG45 Are Essential in Strain JF4278

Protein-coding genes identified as non-essential in PG45 by Sharma et al. (2014) were compared to the results obtained for strain JF4278 with ESSENTIALS. In PG45, 319 independent transposon insertions were sequenced directly from genomic DNA. Based on the presence of at least one transposon insertion located after the first three codons and within the first 85% of the protein-coding sequence, 128 genes were assigned as non-essential in PG45 (Sharma et al., 2014). Using NCBI protein–protein BLAST, 118 out of the 128 non-essential PG45 were found to have homologs in JF4278 (Supplementary Table 4). Concerning the remaining 10 genes, no homolog was found in JF4278 (Supplementary Table 4).

Among these 118 genes, 99 were also found to be non-essential in JF4278 (Supplementary Table 4). Thereby, non-essentiality was confirmed for the vast majority of genes (99/118). However, 19 homologs were differentially assigned as essential in JF4278 compared to non-essential in PG45 (Supplementary Table 4). To assess this discrepancy, these 19 genes were compared to results previously obtained with *M. pulmonis* (Table 2). Ten out of these 19 genes had essential orthologs in *M. pulmonis* strain UAB CTIP (MYPU) (French et al., 2008; Dybvig et al., 2010; Table 2). Six out of the 19 differentially assigned genes were shown to be essential in other *Mycoplasma* spp. and even to belong to the core set of essential genes in *Mycoplasma* genomes (Table 2; Glass et al., 2006; French et al., 2008; Dybvig et al., 2010; Lin and Zhang, 2011; Sharma et al., 2014; Lluch-Senar et al., 2015). Notably, among the non-essential genes identified in the study by Sharma et al. (2014), five genes were found to be essential in all previous gene essentiality studies in mycoplasmas. These five genes (*trmE*, *potB*, *metK*, *dnaJ*, and *smpB*) even belonged to the core set of essential genes in *Mycoplasma* genomes (Lin and Zhang, 2011). However, all of these five genes were found to be essential in *M. bovis* strain JF4278 (Table 2). In fact, *trmE* (MBOVJF4278_00029), *potB* (MBOVJF4278_00110), *metK* (MBOVJF4278_00655), *dnaJ* (MBOVJF4278_00841), and *smpB* (MBOVJF4278_00857) even belonged to the top candidates of JF4278 essential genes identified by ESSENTIALS [log2(FC) value ≤ −8.4, Supplementary Table 2]. Discrepancies in the essentiality assignment of *M. bovis* genes might be due to strain specificity as well as to culture conditions or to mutant library saturation or to the used essentiality analysis method. In consequence, the discrepancies in the essentiality assignment of genes belonging to the core set of essential genes in other *Mycoplasma* spp. should be considered with caution (Table 2). It should be also mentioned that some biological factors can influence the physical accessibility of transposases to some segments of the chromosome. For example, proteins binding DNA and the structure of the chromosomal DNA might modify the frequency of insertion and lead to the identification of false essential genes (Kimura et al., 2016).

**TABLE 2:** Protein-coding genes found as non-essential in PG45 but essential in JF4278.

**Essential CDS in JF4278 (MBOVJF4278_)**	**Product**	**Non-essential PG45 homolog^1^ (MBOVPG45_)**	**PG2 ortholog^2^ (MAG_)**	**Gene persistence^3^**	**Essential UAB CTIP ortholog^4^ (MYPU_)**	**Core mycoplasma genes^5^ (CEMyc_)**
00029	tRNA modification GTPase MnmE	0060	0530	20	0130	0050
00034	50S ribosomal protein L34	0065	7500	20	1540	N/A
00088	Oligopeptide transport ATP-binding protein OppF	0116	1040	15	N/A	N/A
00110	Transport system permease protein PotB	0135	1260	20	4240	0750
00180	Hypothetical protein	0662	N/A	N/A	N/A	N/A
00300	SUA5-like translation suppressor	0534	4520	17	6130	N/A
00382	Potassium transporter trkA	0464	3750	17	1370	N/A
00448	Endonuclease MutS2	0404	3140	1	N/A	N/A
00463	Hypothetical protein	0390	3010	7	7700	N/A
00529	Hypothetical protein	0349	4660	11	N/A	N/A
00561	Putative endonuclease 4	0317	4990	20	6210^∗^	01290
00655	*S*-adenosylmethionine synthase	0227	5800	20	7020	01380
00755	Modification methylase DpnIIB	0766	6680	4	N/A	N/A
00792	Ribosome maturation factor RimP	0800	7000	7	N/A	N/A
00833	Putative RNA pseudouridine synthase	0831	7190	11	N/A	N/A
00841	Chaperone protein DnaJ	0839	7280	12	7330	01460
00846	Phenylalanine-tRNA ligase beta subunit	0845	7310	14	4860	N/A
00857	SsrA-binding protein	0855	7390	20	3520	0650
00865	Catabolite control protein A	0858	7400	5	N/A	N/A

*^1^Non-essential homolog in *M. bovis* PG45 (Sharma et al., 2014). ^2^*M. agalactiae* strain PG2 ortholog. ^3^Gene persistence out of 20 *Mycoplasma* spp. (Liu et al., 2012). ^4^Essential gene in *M. pulmonis* strain UAB CTIP (French et al., 2008). ^5^Core set of essential genes in *Mycoplasma* genomes (Lin and Zhang, 2011). ^∗^Non-essential gene in *M. pulmonis* strain UAB CTIP (Dybvig et al., 2010).*

### Non-essential Genes of *M. bovis* Strain JF4278

Genes identified as non-essential in JF4278 represent a list of individual genes tolerating disruption (Supplementary Table 2). The functional assignment of many *M. bovis* genes is often based on homology predictions and many genes are annotated as hypothetical proteins with unknown function (Supplementary Table 2). Therefore, the list of non-essential JF4278 genes might be useful for future studies aiming to generate *M. bovis* mutants of specific genes for subsequent functional characterization and broaden the knowledge of *M. bovis* biology.

### Several Virulence-Related Factors Identified With the Virulence Factor Database Are Non-essential in *M. bovis* Strain JF4278

To investigate whether putative virulence-related factors are dispensable for *M. bovis*, all the putative proteins of *M. bovis* were analyzed using the Virulence Factor Database (VFDB) (Chen et al., 2016). The hypothetical proteins of *M. bovis* strain JF4278 were aligned against the full dataset of VFDB, containing characterized and predicted virulence-related factors. Seventy-eight genes in the genome of JF4278 were predicted to encode for virulence-related factors based on a BLAST score ≥80 (Supplementary Table 5). In Supplementary Table 5, the gene product names of the VFDB hit are given and not the product names of *M. bovis* strain JF4278. Additionally, previously characterized *M. bovis* virulence-related factors involved in adhesion, cytotoxicity, and H_2_O_2_ production were identified in JF4278 based on a BLAST score ≥80 (Wise et al., 2011; Song et al., 2012; Zou et al., 2013; Sharma et al., 2015; Zhang et al., 2016; Guo et al., 2017; Zhao et al., 2017; Mitiku et al., 2018; Supplementary Table 5). With the VFDB candidates and the known *M. bovis* virulence factors, 91 genes in the JF4278 genome were predicted to encode for virulence-related factors (Supplementary Table 5). As described above, a stricter non-essentiality assignment was applied to the dataset. Forty-six out of the 91 genes were found to have more than one insertion within the core (5–80%) of the gene (Supplementary Table 5). These genes represent putative virulence-related factors that are dispensable for *M. bovis* strain JF4278 growth in rich medium. However, many genes identified via BLAST against the VFDB data base show a very low BLAST score and *E*-value to the *M. bovis* gene and might not be homologs.

Top candidates of non-essential putative virulence-related factors are shown in Table 3 (BLAST score ≥ 200). Additionally, several previously characterized *M. bovis* virulence factors were found to be non-essential: the NADH oxidase (NOX; MBOVJF4278_00281) (Zhao et al., 2017); the TrmFO protein (MBOVJF4278_00132) (Guo et al., 2017), the VpmaX protein (MBOVJF4278_00429) (Zou et al., 2013), and the membrane nuclease MnuA protein (MBOVJF4278_00670) (Sharma et al., 2015; Mitiku et al., 2018; Table 3). Other known *M. bovis* virulence factors like the secretory nuclease (MBOVJF4278_00567; Zhang et al., 2016) or an α-enolase (MBOVJF4278_00443) (Song et al., 2012) were found to be essential or with only few insertions (Supplementary Table 5). The *vsp*-locus of *M. bovis* strain JF4278 comprises several genes (MBOVJF4278_00797–MBOVJF4278_00821) and was identified based on a protein BLAST against PG45 Vsps (Wise et al., 2011). All Vsps identified were found to be non-essential with ESSENTIALS (Supplementary Table 5). Since reads from highly repetitive regions of *vsp*-genes cannot be assigned to a specific position in the genome, they are not used by ESSENTIALS, resulting in *vsp*-genes showing few insertions (Supplementary Table 5). Whether just few insertions are tolerated or the complete *vsp*-locus of strain JF4278 is dispensable remains unknown. Moreover, due to the high frequency of phase and size variation of Vsps, the expressed Vsp forms in the different mutants cannot be predicted (Behrens et al., 1994; Lysnyansky et al., 1996, 1999, 2001).

**TABLE 3:** Non-essential virulence-related factors in M. bovis strain JF4278.

**Non-essential CDS in JF4278 (MBOVJF4278_)**	**VFDB hit and/or *M. bovis* virulence attributes**	**Blast score**	**Blast *E*-value**
00282	IS1634AV transposase [*MmmSC*]	1060	0
00094	IS1634AV transposase [*MmmSC*]	1059	0
00280	IS1634AV transposase [*MmmSC*]	1025	0
00861	IS1634AV transposase [*MmmSC*]	1018	0
00281	NADH oxidase [*M. bovis*]^1^	926	0
00808	Variable surface lipoprotein N [*M. bovis*]^2^; (VpmaVprecursor [*M. agalactiae*])	870; (313)	0; (1*E*−85)
00132	TrmFO [*M. bovis*]^3^	859	0
00670	Membrane nuclease MnuA [*M. bovis*]^4^; (membrane nuclease [*M. pulmonis*])	751; (173)	0; (2*E*−43)
00889	P48, predicted lipoprotein [*M. agalactiae*]	743	0
00150	P48, predicted lipoprotein [*M. agalactiae*]	627	1*E*−180
00700	Endopeptidase Clp ATP-binding chain C [*Listeria monocytogenes*]	546	1*E*−155
00006	Oligopeptide ABC transporter [*MmmSC*]	414	1*E*−116
00429	VpmaX [*M. bovis*]^5^	353	2*E*−126
00819	Variable surface lipoprotein K [*M. bovis*]	289	2*E*−103
00390	PgPepO oligopeptidase [*Mycobacterium* sp. JLS]	280	3*E*−75
00450	Peptide methionine sulfoxide reductase [*Neisseria meningitidis*]	280	8*E*−76
00684	Lipid A ABC exporter [*Haemophilus somnus*]	273	2*E*−73
00453	Lipid A export ATP-binding protein MsbA [*H. influenzae*]	234	1*E*−61
00884	Putative lipoate protein ligase A [*L. ivanovii* subsp. *ivanovii*]	229	2*E*−60
00797	Variable surface lipoprotein H [*M. bovis*]^2^	215	7*E*−74
00803	Variable surface lipoprotein G [*M. bovis*]^2^	201	4*E*−66

*Non-essential virulence-related proteins identified by VFDB for *M. bovis* JF4278 using BLAST. Known *M. bovis* virulence-related factors were identified by BLAST. ^1^NADH oxidase (Zhao et al., 2017). ^2^Variable surface lipoproteins (Wise et al., 2011). ^3^TrmFO (Guo et al., 2017). ^4^Membrane nuclease MnuA (Sharma et al., 2015; Mitiku et al., 2018). ^5^VpmaX (Zou et al., 2013). Scores and *E*-values were generated by the BLAST algorithm. VFDB hits were filtered by BLAST scores >200.*

The exact function of putative virulence-related factors identified via VFDB in *M. bovis* remains speculative (Table 3). However, some notable factors were characterized in previous studies. The P48 lipoprotein (MBOVJF4278_00889 and _00150) stimulates the host humoral response in *M. agalactiae* (Table 3; Rosati et al., 2000). The endopeptidase Clp ATP-binding chain C (ClpC; MBOVJF4278_00700) is required for *Listeria monocytogenes* adhesion and invasion (Nair et al., 2000). Additionally, it promotes early escape from the phagosome of macrophages and intracellular survival by modulation of other factors at the transcription level (Rouquette et al., 1998; Nair et al., 2000; Table 3). Similarly, the lipoate protein ligase A (LplA1; MBOVJF4278_00884) was found to be necessary for efficient intracellular proliferation of *L. monocytogenes* (O’Riordan et al., 2003; Table 3). The PgPepO oligopeptidase (Zmp1; MBOVJF4278_00390) allows *Mycobacterium tuberculosis* survival in macrophages by interfering with phagosome maturation and inhibiting the formation of the inflammasome (Paolino et al., 2018; Table 3). The peptide methionine sulfoxide reductase (MsrA; MBOVJF4278_00450) might be involved in the protection from oxidative damage through exogenous as well as endogenous oxidants in *Neisseria meningitidis* and *M. genitalium* and adhesion in *M. genitalium* (Dhandayuthapani et al., 2001; Olry et al., 2002; Table 3). All these genes could have a potential role in the virulence of *M. bovis.* However, functional studies coupled with *in vitro* and *in vivo* infections are necessary to confirm or infirm these hypotheses. The identification of non-essential virulence-related factors could also lead to the design and generation of attenuated *M. bovis* strains for subsequent vaccine development. Many factors included in the VFDB are clearly linked to virulence and are well characterized as virulence attributes in the respective species. However, a clear and direct link to virulence is not obvious for all factors present in this database. For example, transposases and other mobile genetic elements are widely distributed in *Mycoplasma* spp. and are involved in genetic plasticity of genomes and horizontal gene transfer (Sirand-Pugnet et al., 2007a, b; Lysnyansky et al., 2009; Table 3). Indeed, insertion sequences and transposases can transfer antibiotic-resistant genes and genes encoding for virulence factors and indirectly impact the virulence of strains (Vandecraen et al., 2017; Partridge et al., 2018).

### A Set of *M. bovis* Genes Are Underrepresented After the Adhesion Assay, These Conditional-Essential Genes Might Be Adhesion-Related Factors

**FIGURE 5:**
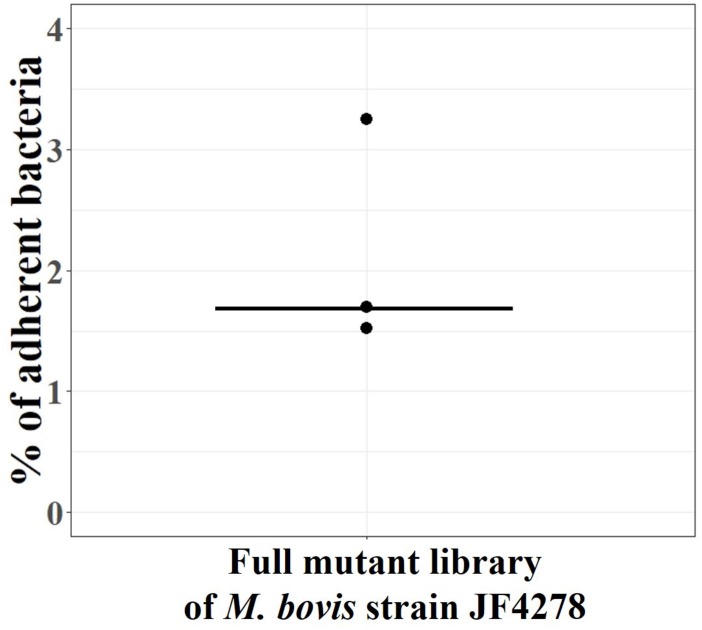
Adhesion assay with the full *M. bovis* strain JF4278 mutant library. *M. bovis* adhesion to bMec cells after 30 min. The *y*-axis represents the percentage of adherent *M. bovis* relative to the added *M. bovis*, calculated by using the number of CFUs counted from input samples and output samples. Adhesion assay with the full *M. bovis* mutant library was performed in three independent experiments indicated by black dots. Median values are indicated as horizontal lines.

The full *M. bovis* strain JF4278 mutant library was screened for adhesion (Figure 1). The percentage of adherent *M. bovis* (output samples) relative to the total amount of mycoplasma cells used for infection (input samples) was calculated based on CFUs counted from input and output samples. The median value for mycoplasma adhesion to bMec cells from the three independent replicate experiments was 1.7% (Figure 5). Approximately, 5 × 10^7^ mycoplasmas (CFU) including all the 4000 mutants were used in each replicate of the adhesion assay. With a median adhesion value of 1.7%, approximately 2 × 10^2^ cells per mutant were recovered from the assay in each of the replicates. As mutants deficient in adhesion are expected to be more efficiently washed away during the adhesion assay, they are underrepresented in the output samples compared to other mutants. Based on this assumption, output samples were compared to input samples to identify conditional-essential genes (genes identified as non-essential for growth but essential for adhesion).

**FIGURE 6:**
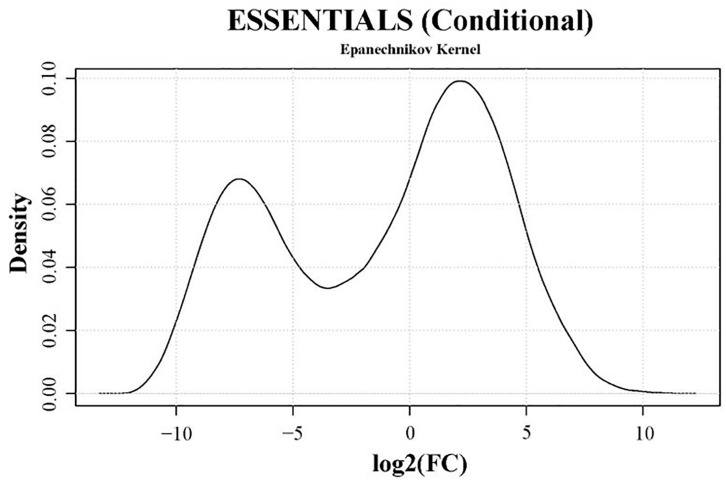
Density plot of conditional-essentiality values obtained with the software ESSENTIALS. Read counts per gene of output samples were compared to data from input samples by ESSENTIALS (Zomer et al., 2012). *M. bovis* strain JF4278 genes from output samples are assigned a fold change (FC) value of read counts in log2 scale compared to the read counts of input samples. Log2(FC) values for all genes are shown on the *x*-axis of the density plot. Log2(FC) values show a bimodal distribution. The log2(FC) value of non-essential JF4278 genes is around zero. Genes more likely to be conditional-essential (essential for adhesion) have negative log2(FC) values. The cut-off value for conditional-essential genes suggested by the software ESSENTIALS for this data set is log2(FC) < –3.38.

The transposon-junctions of mutants recovered from the adhesion assay (output samples) were sequenced. Transposon sequencing data from input and output samples were used for the conditional-essentiality analysis using ESSENTIALS to identify genes putatively involved in adhesion. The read counts per gene of output samples were compared to data from input samples by ESSENTIALS. Thereby, genes from output samples are assigned a FC value of read counts in log2 scale (Figure 6 and Supplementary Table 6). The log2(FC) values of JF4278 genes after the adhesion assay show a bimodal distribution in the density plot (Figure 6). The log2(FC) value of non-essential genes for growth and for adhesion is around zero (Figure 6). On the other hand, genes with underrepresented read counts after the adhesion assay [strongly negative log2(FC) values] are more likely to be conditional-essential (Figure 6). Significantly underrepresented (adj. *P*-value ≤ 0.05) genes below an automatically calculated log2(FC) cut-off value were considered as conditional-essential genes (Zomer et al., 2012). The log2(FC) cut-off for conditional-essential genes suggested by ESSENTIALS was <−3.38 (Figure 6).

**FIGURE 7:**
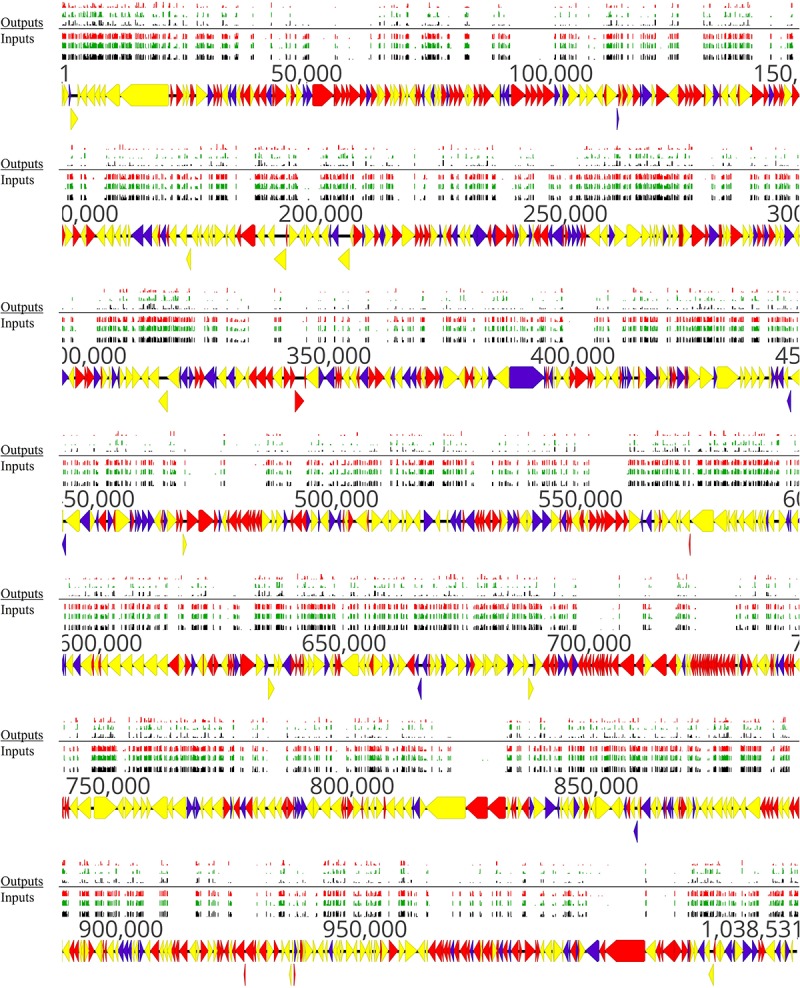
Overview of non-essential, essential, and conditional-essential genes in the genome of *M. bovis* strain JF4278. Genes and their orientation are indicated by arrows. Genes found essential with ESSENTIALS are filled in red, while non-essential genes are filled in yellow (Zomer et al., 2012). Genes found to be conditional-essential (essential for adhesion) are filled in purple. Essentiality was assessed with transposon sequencing data of the full mutant library (input samples) with ESSENTIALS. Conditional-essentiality was assessed by comparing input samples to output samples with ESSENTIALS. Mapping of the transposon integrations sites from the transposon sequencing of the input samples (Inputs) and output samples (Outputs) are represented by black, green, and red bars (each corresponding to one replicate). The height of the insertion bars represents the read count per insertion.

According to these criteria [log2(FC) < −3.38 and adj. *P*-value ≤ 0.05], 196 out of 548 non-essential genes were found to be conditional-essential (Supplementary Table 6). On the other hand, transposon sequencing reads from 66 genes were significantly overrepresented after the adhesion assay (Supplementary Table 6). Distribution of transposon insertion sites as well as the read counts in log10 scale (height of bars) from the three sequencing replicates of the full mutant library (input samples) and of the mutants recovered after the adhesion assay (output samples) are in line with the predicted conditional-essentiality of genes (Figure 7 and Supplementary Table 6). Genes with fewer transposon insertions and smaller numbers of reads in the output samples (Figure 7, Outputs) compared to input samples (Figure 7, Inputs) were identified as conditional-essential (Figure 7, purple arrows). In addition to the prediction of adhesion-related factors using ESSENTIALS, SPAAN analysis of the predicted proteome of *M. bovis* was performed (Sachdeva et al., 2005). Putative proteins were assigned a *P*_ad_ value reflecting the probability of a protein to be an adhesin, with higher values reflecting higher probabilities (Supplementary Table 6).

The set of genes identified to be conditional-essential represents a list of potential adhesion-related factors in *M. bovis*. For example, the NADH oxidase (MBOVJF4278_00281) and the TrmFO protein (MBOVJF4278_00132), which are known *M. bovis* adhesins (Guo et al., 2017; Zhao et al., 2017), were predicted to be conditional-essential and involved in adhesion (Supplementary Table 6). Endopeptidase Clp ATP-binding chain C (MBOVJF4278_00700) identified with the VFDB and known to be involved in adhesion in *L. monocytogenes* (Nair et al., 2000; Table 3) was assigned as a putative adhesion-related factor in *M. bovis* (Supplementary Table 6). However, ESSENTIALS failed to predict most of the Vsps and VpmaX (MBOVJF4278_00429) to be conditional-essential in the adhesion assay (Supplementary Table 6). This is probably due to the repetitive regions of *vsp*-genes. Indeed, sequence reads from repetitive regions cannot be assigned to a specific position in the genome and are not considered by ESSENTIALS. Moreover, the *M. bovis* adhesion factor α-enolase (MBOVJF4278_00443) previously shown to be essential could not be assigned to a conditional-essential phenotype (Song et al., 2012; Supplementary Tables 2, 6).

The combined information from adhesion-related factors identified by ESSENTIALS and SPAAN is useful to identify candidate genes for future adhesion studies (Supplementary Table 6). Nevertheless, false positives cannot be ruled out and therefore candidate mutants for adhesion should be individually tested. As mentioned above, approximately 2 × 10^2^ cells per mutant were recovered from the adhesion assay in each of the replicates. This rather small number of recovered cells per mutant represents a potential bottleneck during the adhesion assay. Especially mutants that grew slowly during the collection of individual mutants and are underrepresented in the full mutant library, even before the infection, could lead to false positive adhesion candidates. To prevent a potential bottleneck during the adhesion assay, the number of different mutants used could be reduced to recover more than 2 × 10^2^ cells per mutant after adhesion. However, including fewer mutants in this study would have hampered the accurate assignment of essential and non-essential genes from input samples. For these reasons, conditional-essential genes with the lowest log2(FC) values (Supplementary Table 6) and with the highest adhesion probability values by SPAAN (Supplementary Table 6) are top candidate genes for adhesion to be tested in future studies.

### Three Individual Mutants Showed a Reduced Adhesion Phenotype

Six candidate mutants from the complete spectrum of putative adhesion-related factors identified by ESSENTIALS and SPAAN were selected to be individually evaluated for adhesion: MBOVJF4278_00132, MBOVJF4278_00255, MBOVJF4278_ 00264, MBOVJF4278_00598, MBOVJF4278_00667, and MBOVJF4278_00812 (Table 4). A mutant of the gene coding for the TrmFO protein (MBOVJF4278_00132) was selected because this protein was previously characterized as an adhesin in *M. bovis* (Guo et al., 2017), despite the fact that it was not among the top candidates identified with ESSENTIALS and SPAAN analysis (Table 4 and Supplementary Table 6). According to TMpred TrmFO displays one putative TM-H with a score of 424 which is just below the suggested cut-off value of 500. The hypothetical protein MBOVJF4278_00255 is a homolog of the putative lipoprotein MBOVPG45_0586 of PG45 that belongs to the LppB family. MBOVJF4278_00255 was among the top candidates for adhesion identified with ESSENTIALS and SPAAN and was predicted to contain two TM-Hs (Table 4 and Supplementary Table 6). The hypothetical protein MBOVJF4278_00264 is a homolog of the putative lipoprotein MBOVPG45_0574 in PG45 and belongs to the PARCEL family. MBOVJF4278_00264 was identified with ESSENTIALS and SPAAN as an intermediate candidate for adhesion and was predicted to contain one TM-H (Table 4 and Supplementary Table 6). The putative phosphatase MBOVJF4278_00598 is a homolog of MBOVPG45_0281 in PG45 and belongs to the HAD-superfamily hydrolase, subfamily IIB. Based on SPAAN and ESSENTIALS, this protein displayed a high probability to be involved in adhesion but no TM-H was predicted (Table 4 and Supplementary Table 6). The hypothetical protein MBOVJF4278_00667 was also among the top candidates for adhesion identified with ESSENTIALS. However, the SPAAN value was notably low (Table 4 and Supplementary Table 6). The TMpred software predicted one TM-H for this protein (Table 4). MBOVJF4278_00812, encoding for a hypothetical protein, was the only gene significantly underrepresented from the *vsp*-locus (Supplementary Table 6). The sequence obtained from the genome of JF4278 indicates that MBOVJF4278_00812 is a truncated form of the VspN in PG45 (MBOVPG45_0811). Even though adhesion-prediction by ESSENTIALS and SPAAN was low (Table 4 and Supplementary Table 6), MBOVJF4278_00812 was included as a candidate for adhesion in agreement with the Vsps being previously shown to be involved in adhesion (Sachse et al., 1993, 1996, 2000; Thomas et al., 2003).

**TABLE 4:** Features of the M. bovis potential adhesion-deficient candidate mutants.

**Candidate mutant (MBOVJF4278_)**	**Product**	**Tn insertion site in gene (%)**	**ESSENTIALS log2(FC)^1^**	**Adhesion probability SPAAN^2^**	**TM-helices^3^**
00132	TrmFO	50.8	−4.0	0.23	0
00255	Hypothetical protein	19.8	−8.7	0.71	2
00264	Hypothetical protein	65.2	−7.9	0.48	1
00598	Putative phosphatase MPN_383	61.8	−8.1	0.70	0
00667	Hypothetical protein	25.6	−9.2	0.26	1
00812	Hypothetical protein	66.4	−5.0	0.54	1

*^1^Conditional-essentiality log2(FC) value by ESSENTIALS (Zomer et al., 2012). ^2^Adhesion probability value by SPAAN (Sachdeva et al., 2005). ^3^Number of transmembrane helices predicted by TMpred scores >500.*

**FIGURE 8:**
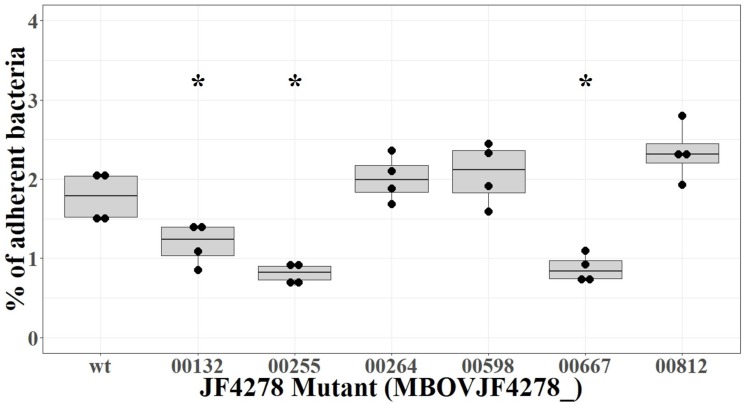
Adhesion assay of individual candidate mutants of *M. bovis* strain JF4278. *M. bovis* adhesion to bMec cells after 30 min. The *y*-axis represents the percentage of adherent *M. bovis* relative to the added *M. bovis*. Adhesion with the wild-type strain or single mutants was performed in four independent experiments as indicated by black dots. Median values are indicated as horizontal lines in the box plot. Statistical comparison of each mutant to the wild-type strain was performed by the two-sample Wilcoxon test. ^∗^*P* < 0.05.

Adhesion of the individual *M. bovis* mutants to bMec cells was compared to wt strain JF4278. The median adhesion was 1.8% for the wt strain JF4278 (Figure 8). Three out of six mutants showed significantly lower adhesion capacity to bMec cells compared to the wt: a mutant of the gene coding for the TrmFO protein (MBOVJF4278_00132) and mutants of two genes coding for hypothetical proteins (MBOVJF4278_00255 and MBOVJF4278_00667) (Figure 8). The MBOVJF4278_00132 mutant showed a median adhesion of 1.2% (Figure 8). Both of the two newly identified adhesion-related factors had a median adhesion of 0.8%, which is over 50% lower than the wt strain JF4278 (Figure 8). The median adhesion value of the other three selected mutants, MBOVJF4278_00264, MBOVJF4278_00598, and MBOVJF4278_0081, was not significantly different from the wt strain (Figure 8).

MBOVJF4278_00255 and MBOVJF4278_00667 were among the top candidates identified with ESSENTIALS (Supplementary Table 6). Moreover, the presence of at least one TM-H was predicted for both genes (Table 4). MBOVJF4278_00255 was also among the top candidates for adhesion by the SPAAN analysis (Supplementary Table 6). Therefore, a focus on top candidates and a combination of the results obtained with ESSENTIALS, SPAAN, and TMpred could improve the prediction of putative adhesins. Nevertheless, it is crucial to test single mutants to confirm results obtained via high-throughput approaches. The final assignment of an adhesin should then include experiments to complement mutants. Moreover, the surface exposure of these putative adhesins should be characterized *in vitro*. An additional point to consider is the expression of Vsps in the wt strain and in mutants. As the *vsp*-locus undergoes recombination events, the expression of Vsps might differ between a mutant and the wt strain, potentially affecting adhesion.

## Conclusion

Addressing gene essentiality is always dependent on the experimental settings, in particular the conditions used for preparing and propagating mutant libraries and the analysis method chosen. In recent years, methods to assign essentiality from transposon mutant libraries have developed, from looking at whether a gene was disrupted by a transposon, to applying rules for disruption, to high-throughput analysis approaches involving statistics for essentiality probabilities (Christen et al., 2011; Zomer et al., 2012; Lluch-Senar et al., 2015; Solaimanpour et al., 2015). Similar to ESSENTIALS, these methods also require high-density mutant libraries where few transposon insertions can be detected in essential genes, but this is counterbalanced by taking into account the amount of associated reads. Additionally, one recurrent question is whether the simultaneous disruption of non-essential genes identified by transposon mutagenesis results in viable bacteria and whether some mutants are mutually exclusive (Hutchison et al., 2016). Data collected in this study resulted in proposing a list of individual *M. bovis* genes tolerating disruption among which several non-essential putative virulence-related factors were identified. Moreover, three clones showed reduced bovine cell adhesion. Overall, datasets of non-essential and conditional-essential genes are both a source of valuable information for further exploring *M. bovis* biology, pathogenesis, and interaction with the host as well as for vaccine design.

## Data Availability

The transposon sequencing datasets analyzed for this study can be found in the ENA (http://www.ebi.ac.uk/ena/data/view/PRJEB31021).

## Author Contributions

CJ, SB, ED-F, CC, LF, and PP designed the experiments. CJ, SB, and SV performed the experiments. CJ, LF, and PP analyzed the data. CJ drafted the manuscript. All authors helped in writing the manuscript and critically revised it. All authors read and approved the final manuscript.

## Conflict of Interest Statement

The authors declare that the research was conducted in the absence of any commercial or financial relationships that could be construed as a potential conflict of interest.

## Footnotes

1 https://usegalaxy.org

2 http://bamics2.cmbi.ru.nl/websoftware/essentials/essentials_start.php

3 http://kobas.cbi.pku.edu.cn/index.php

4 https://www.genome.jp/kegg/

5 https://blast.ncbi.nlm.nih.gov/Blast.cgi

6 https://tubic.org/pdeg/core.php?page=1

7 https://www.wessa.net/rwasp_density.wasp

8 https://embnet.vital-it.ch/software/TMPRED_form.html
