# Supplementary material for: Large-Scale Analysis of the Mycoplasma bovis Genome Identified Non-essential, Adhesion- and Virulence-Related Genes
Source: Front Microbiol. 2019 Sep 13;10:2085. doi: 10.3389/fmicb.2019.02085 (PMC6753880; doi:10.3389/fmicb.2019.02085)
Supplement: Supplementary file 1 [file Table_1.DOCX]

Supplementary Table 1: Supplementary Methods

# Supplementary Methods

Primers for the amplicon library preparation are listed in **Supplementary Methods Table A** (below). Alignment parameters are shown in **Supplementary Methods Table B** (below). Parameters used for the essentiality analysis by ESSENTIALS are shown in **Supplementary Methods Table C** (below). Primers used for the detection of candidate mutants are shown in **Supplementary Methods Table D** (below).

# DNA library preparation for transposon sequencing on the Illumina NextSeq Platform

The input and the output DNA samples of the mutant library were prepared for transposon sequencing on the Illumina NextSeq Platform. Two major steps were performed: 1) Enrichment of amplicons from the transposon-junctions and depletion of eukaryotic DNA and 2) Amplification of the library to add Illumina adapters. Tn*4001*‑junction sequences from the genomic DNA of the input and output samples were enriched by linear PCR using the biotinylated transposon specific primer T1_Biotin (**Supplementary Methods Table A**) as previously described with slight modifications (Paruzynski et al., 2010). Briefly, *Pfu* polymerase (Genaxxon bioscience GmbH, Ulm, Germany) was used and the extension time was adjusted to 35 sec (Paruzynski et al., 2010). To amplify the collected DNA samples, 3 or 10 separate PCR reactions had to be performed for input and output samples, respectively. Linear PCR products of each sample were pooled and concentrated with the GeneJET PCR Purification Kit (Thermo Fisher Scientific) according to the manufacturer’s protocol. To prevent saturation, two columns were used to extract DNA from output samples. DNA from the same sample was pooled after elution in a total volume of 150 µL elution buffer. The concentration and purity of the DNA samples were checked with a NanoDrop 1’000 spectrophotometer (Thermo Fisher Scientific). The first magnetic capture using Dynabeads M-280 streptavidin (Thermo Fisher Scientific) was performed following a previously established protocol with LiCl washing steps and a 15 h incubation step at room temperature (Paruzynski et al., 2010). After incubation, the DNA-beads complex was washed twice with 500 µL ddH_2_O. A deoxycytosine homopolymer tail (C-tail) of controlled length using Terminal Deoxinucleotidyl Transferase (TdT) (Thermo Fisher Scientific) was added to the linear amplification products directly on the beads following a protocol described by Lazinski and Camilli (2013). After heat inactivation of TdT, the DNA‑beads complex was washed twice with 500 µL ddH_2_O to get rid of potential residuals of ddCTP (Genaxxon bioscience GmbH) and was then eluted in 10 µL ddH_2_O. Subsequent exponential PCR reactions were performed using *TaKaRa Ex Taq* DNA polymerase (Takara Bio Europe SAS, Saint-Germain-en-Laye, France) (Dawoud et al., 2014). Elongation time of the exponential PCRs was adjusted to 10 sec compared to the protocol published by Paruzynski et al. (2010). The first exponential PCR was performed using a nested biotinylated transposon specific primer (T3_Biotin) and a C-tail specific linker primer (PolyG_Linker) (**Supplementary Methods Table A**). To amplify the complete DNA from each sample, two separate PCR reactions were performed per sample. The PCR products of each input or output sample were pooled after cycling. The presence of the specific DNA smear after the first exponential PCR was confirmed on an agarose gel for input and output DNA samples recovered from the JF4278 mutant library. The absence of any PCR product after the first exponential PCR was confirmed on an agarose gel for input and output DNA samples of the wild type strain JF4278. Magnetic capture of the first exponential PCR product for 2.5 h at room temperature and subsequent denaturation of dsDNA were performed as described by Paruzynski et al. (2010). A second exponential PCR was performed using a nested transposon specific primer (NGS_1st_PCR_fwd) and a primer specific to the linker added in the 1^st^ exponential PCR reaction (NGS_1st_PCR_rev). Both NGS primers contain five wobble bases between the locus specific sequence and the universal 5’ tail as specified in the Nextera library protocol from Illumina (**Supplementary Methods Table A**). Three separate PCR reactions were performed per sample. The PCR products of each input or output sample were pooled after cycling. The presence of the specific DNA smear after the second exponential PCR was confirmed on an agarose gel for input and output DNA samples recovered from the JF4278 mutant library. The absence of any PCR product after the second exponential PCR using either the reverse or forward primer alone was confirmed on an agarose gel for input and output samples. Pooled PCR products were bead-purified using the Agencourt AMPure XP kit (Beckman Coulter International S.A., Nyon, Switzerland) according to the manufacturer’s protocol. DNA concentrations were measured with the Qubit 3.0 Fluorometer (Thermo Fisher Scientific) using the Qubit Assay Kit (dsDNA HS Assay) according to the manufacturer’s protocol. Before sequencing, the input and output samples were sent to Microsynth (Microsynth AG, Balgach, Switzerland) for further preparation. Microsynth barcoded the samples in a final PCR and the Illumina adapters were added. The six libraries were gel purified, quantified, pooled and sequenced in the same run with Illumina NextSeq 1 x 75 bp high-output.

**TABLE A | Primers for amplicon library preparation**

| **Primer Name** | **Oligonucleotide sequence (5'->3')** | **Modifications** | **Function** |
| --- | --- | --- | --- |
| T1_Biotin | CTGATTCTGTGGATAACCGTATTACCGCCTTTG | 5' biotinylated | Amplicon library preparation pMT85-Tet transposon specific |
| T3_Biotin | GTTGGCCGATTCATTAATGCACGCTAGC | 5' biotinylated | Amplicon library preparation pMT85-Tet transposon specific |
| PolyG_Linker | TCAGTGGCACAGCAGTTAGGGGGGGGGGGG | 5' Linker | Amplicon library preparation C-tail specific |
| NGS_1st_PCR_fwd | TCGTCGGCAGCGTCAGATGTGTATAAGAGACAGNNNNNACCCTTTTACACAATTATACGGACT | 5' Linker for Nextera library protocol and five wobble bases | Amplicon library preparation pMT85-Tet transposon specific |
| NGS_1st_PCR_rev | GTCTCGTGGGCTCGGAGATGTGTATAAGAGACAGNNNNNAGTGGCACAGCAGTTAGG | 5' Linker for Nextera library protocol and five wobble bases | Amplicon library preparation PolyG-Linker specific |

**TABLE B | Parameters used for Bowtie2 on Galaxy (the adapted Galaxy parameter file is shown) (Langmead and Salzberg, 2012)**

| **Input Parameter** | **Value** |
| --- | --- |
| -U (unpaired reads (single library) in fastq format) | Trimmed fastq files |
| -x (reference genome from history) | GCA_900088685.1_JF4278_genomic.fna Normalized (length 80) |
| --skip (skip that many reads) | 0 (default=0) |
| --qupto (align that many reads, keep number high) | 100000000 |
| --trim5 (trim read from 5') | 0 (default=0) |
| --trim3 (trim read from 3') | 0 (default=0) |
| --phred33 (select quality score from Illumina) | --phred33 |
| -N (number of mismatches allowed in a seed) | 0 (default=0) |
| -L (lenght of seed substrings to align) | 22 (default=22) |
| -i (function for interval between seed substrings) | S,1,1.15 (default=S,1,1.15) |
| --n-ceil (number of ambiguous characters allowed) | L,0,0.15 (default=L,0,0.15) |
| --dpad | 15 (default=15) |
| --gbar | 4 (default=4) |
| --end-to-end (select between local and end-to-end alignment mode) | --end-to-end |
| --score-min (minimum alignment score to be valid) | L,-0.6,-0.6 (default=L,-0.6,-0.6 in end-to-end mode) |
| -D (attempts for seed extension) | 15 (default=15) |
| -R (re-seeds) | 2 (default=2) |

**TABLE C | Parameters used for ESSENTIALS (Zomer et al., 2012)**

| **Input Parameter** | **Value** |
| --- | --- |
| use TA for mariner, random for TN5. | random |
| Library size | 20000 |
| Perform repeat filtering on genome and on matched sequence reads | Yes |
| Barcode mismatch allowed | 0 |
| Genomic sequence remaining of read after removal of barcode and transposon | 25 |
| Select barcode side, eol (end of line) for 3' end, bol (beginning of line) for 5' end | bol |
| Select transposon inverted repeat side, eol (end of line) for 3' end, bol (beginning of line) for 5' end | bol |
| Minimal sequence match required for alignment | 22 |
| Strand to align with: 0=reverse, 1=forward, 2=both | 2 |
| Use 3' truncated genes for matching insertion sites (recommmended) Use truncated.ptt for truncation, for full genes use genome.ptt | genome.ptt |
| Remove genomic position bias using Loess? | Yes |
| TMM, RLE, Quantile or total read count normalization | TMM |
| unpaired (qCML) or paired analysis (Cox-Reid) | qCML |
| Modeling of variance, common or tagwise dispersion | tagwise |
| Prior.n: Amount of smoothing of tagwise dispersion | 20 |
| P-value adjustment methods | BH |
| P-value | Corrected |
| Minimum number of reads | 1 |
| Create ZIP archive | Yes |
| Email | christoph.josi@vetsuisse.unibe.ch |

**TABLE D | Primers for the detection of the selected mutants**

| **Mutated gene** | **Tn insertion position in genome (direction)** | **Tn insertion sites in gene (%)** | **Primer Name** | **Olignonucleotide sequence (5'->3')** |
| --- | --- | --- | --- | --- |
| MBOVJF4278_00132 | 156731 (-) | 51 | 00157 | GCAATAGTTTCAATTGAACTAAAGCC |
| MBOVJF4278_00255 | 295439 (-) | 20 | 00280 | GAGCACAATCTGGTGCTTATG |
| MBOVJF4278_00264 | 306406 (+) | 65 | 00289 | CAGTTTCCAAAAACATTTTGTGC |
| MBOVJF4278_00598 | 719417 (+) | 62 | 00625 | CCGTACAAACACACAGCAATTTC |
| MBOVJF4278_00667 | 789053 (-) | 26 | 00694 | CCTTGCTTGATGGTTTTGGG |
| MBOVJF4278_00812 | 942859 (-) | 66 | 00835_1 | CCTTGTTCTCCTCTATGTACTCATTCC |
| pMT85-Tet transposon specific | N/A | N/A | SG8 | GAGTCAGTGAGCGAGGAAGC |

# References

Dawoud, T.M., Jiang, T., Mandal, R.K., Ricke, S.C., and Kwon, Y.M. (2014). Improving the Efficiency of Transposon Mutagenesis in *Salmonella* Enteritidis by Overcoming Host-Restriction Barriers. *Molecular Biotechnology* 56(11)**,** 1004-1010. doi: 10.1007/s12033-014-9779-4.

Langmead, B., and Salzberg, S.L. (2012). Fast gapped-read alignment with Bowtie 2. *Nat. Methods* 9(4)**,** 357-359. doi: 10.1038/nmeth.1923.

Lazinski, D.W., and Camilli, A. (2013). Homopolymer tail-mediated ligation PCR: a streamlined and highly efficient method for DNA cloning and library construction. *Biotechniques* 54(1)**,** 25-34. doi: 10.2144/000113981.

Paruzynski, A., Arens, A., Gabriel, R., Bartholomae, C.C., Scholz, S., Wang, W., et al. (2010). Genome-wide high-throughput integrome analyses by nrLAM-PCR and next-generation sequencing. *Nat. Protoc.* 5(8)**,** 1379-1395. doi: 10.1038/nprot.2010.87.

Zomer, A., Burghout, P., Bootsma, H.J., Hermans, P.W.M., and van Hijum, S.A.F.T. (2012). ESSENTIALS: Software for Rapid Analysis of High Throughput Transposon Insertion Sequencing Data. *PLoS One* 7(8)**,** e43012. doi: 10.1371/journal.pone.0043012.
